# AGENCY IN LATE-CAREER TRANSITIONS: THE IMPACT ON ADJUSTMENT AND SATISFACTION

**DOI:** 10.1093/geroni/igz038.077

**Published:** 2019-11-08

**Authors:** van Solinge

**Affiliations:** Netherlands Interdisciplinary Demographic Institute, The Hague, Netherlands

## Abstract

In research on late career transitions agency is implicitly assumed. The extent to which older adults are able to shape their late career in the face of external constraints, such as a rising state pension age, may however be limited. Constraint agency may have impact on well-being. Using data from a panel study among 5,300 older workers in the Netherlands, we examined the impact of agency in the work-retirement transition on adjustment and life satisfaction. Results show that adjustment to a the higher retirement age is more challenging than adjustment to retirement. Life satisfaction increased among those who retired, but not among those who remained working. One third experienced constrained agency (involuntary retirement or non-retirement). The negative association between constrained agency and life satisfaction was stronger for participants still in the labor force than for retirees. Our findings demonstrate that involuntary non-retirement has stronger implications for well-being than involuntary retirement.

